# Plasmid pP62BP1 isolated from an Arctic *Psychrobacter* sp. strain carries two highly homologous type II restriction-modification systems and a putative organic sulfate metabolism operon

**DOI:** 10.1007/s00792-012-0435-2

**Published:** 2012-03-04

**Authors:** Robert Lasek, Lukasz Dziewit, Dariusz Bartosik

**Affiliations:** Department of Bacterial Genetics, Faculty of Biology, Institute of Microbiology, University of Warsaw, Miecznikowa 1, 02-096 Warsaw, Poland

**Keywords:** *Psychrobacter* sp. DAB_AL62B, Plasmid pP62BP1, Restriction-modification systems, Alkylsulfatase, Metabolism of organic sulfates

## Abstract

The complete nucleotide sequence of plasmid pP62BP1 (34,467 bp), isolated from Arctic *Psychrobacter* sp. DAB_AL62B, was determined and annotated. The conserved plasmid backbone is composed of several genetic modules, including a replication system (REP) with similarities to the REP region of the iteron-containing plasmid pPS10 of *Pseudomonas syringae*. The additional genetic load of pP62BP1 includes two highly related type II restriction-modification systems and a set of genes (*slfRCHSL*) encoding enzymes engaged in the metabolism of organic sulfates, plus a putative transcriptional regulator (SlfR) of the AraC family. The pP62BP1 *slf* *locus* has a compact and unique structure. It is predicted that the enzymes SlfC, SlfH, SlfS and SlfL carry out a chain of reactions leading to the transformation of alkyl sulfates into acyl-CoA, with dodecyl sulfate (SDS) as a possible starting substrate. Comparative analysis of the nucleotide sequences of pP62BP1 and other *Psychrobacter* spp. plasmids revealed their structural diversity. However, the presence of a few highly conserved DNA segments in pP62BP1, plasmid 1 of *P. cryohalolentis* K5 and pRWF-101 of *Psychrobacter* sp. PRwf-1 is indicative of recombinational shuffling of genetic information, and is evidence of lateral gene transfer in the Arctic environment.

## Introduction

In recent years, microorganisms adapted to extremely cold environments have become an area of growing scientific interest. This is mainly due to the fact that they represent a likely source of cellular products with potential biotechnological applications, such as cold-adapted enzymes (Cavicchioli et al. 2002). There is an increasing requirement for the development of efficient genetic systems that are functional at low temperatures for the expression and purification of these proteins as well as other heterologous proteins characterized by their heat lability, toxicity towards the host cell or tendency to form inclusion bodies in standard expression hosts (Werbowy et al. 2009). The development of such genetic tools is tightly linked with genomic studies on psychrophilic strains, and especially their indigenous extrachromosomal replicons. To date, a relatively small number of plasmids residing in psychrophilic strains have been characterized (e.g. Cieśliński et al. 2008). This may explain why only a few low temperature expression systems are currently available (e.g. Zhao et al. 2011; Parrilli et al. 2008; Miyake et al. 2007).

In this study, we analyzed the genetic organization of plasmid pP62BP1 of psychrophilic *Psychrobacter* sp. DAB_AL62B. This strain was isolated from guano sediments collected from a breeding colony of little auks (*Alle alle*) in the vicinity of the Polish Polar Station, situated in the Norwegian Svalbard archipelago (77°0′0″N, 15°33′0″E) in the Arctic.

Psychrophilic bacteria of the genus *Psychrobacter* successfully colonize diverse ecological niches in permanently cold or even permafrost regions, as well as in other low water activity environments (Rodrigues et al. 2009). Current knowledge concerning the pool of mobile DNA of *Psychrobacter* spp. is extremely limited. Complete nucleotide sequences of only five plasmids have been obtained to date: pRWF101 and pRWF102 of *Psychrobacter* sp. PRwf-1, pTAUp and pTADw of *Psychrobacter* sp. TA144, and plasmid 1 of *P. cryohalolentis* K5. The largest replicons, pRWF101 (13.9 kb) and plasmid 1 of *P. cryohalolentis* K5 (41.2 kb), contain 14 and 44 open reading frames (ORFs), respectively, while the other plasmids are small (1.3–2.1 kb) cryptic replicons, encoding one or two ORFs. An in-depth sequence analysis has only been reported in the case of the plasmids harbored by the strain TA144 (Tutino et al. 2000; Duilio et al. 2001).

The detailed genomic analysis of plasmid pP62BP1 of *Psychrobacter* sp. DAB_AL62B presented in this report includes the characterization of a variety of genetic modules that may be of great value in the construction of genetic cassettes to be used in the creation of versatile vectors functional in psychrophilic bacteria.

## Materials and methods

### Bacterial strain and culture conditions

The strain DAB_AL62B isolated from little auk guano collected from Spitsbergen Island in the Arctic was classified as a member of the *Psychrobacter* genus due to 16S rRNA sequence similarity. The strain was cultured in LB (lysogeny broth) medium (Sambrook and Russell 2001) at 21°C.

### Plasmid DNA isolation

Plasmid DNA was isolated using a large-scale alkaline extraction method and purified by CsCl-ethidium bromide gradient centrifugation (Sambrook and Russell 2001).

### DNA sequencing

The complete nucleotide sequence of plasmid pP62BP1 was determined in the DNA Sequencing and Oligonucleotide Synthesis Laboratory (oligo.pl) at the Institute of Biochemistry and Biophysics, Polish Academy of Sciences. High-throughput sequencing of the MID-tagged shotgun plasmid-library was performed using an FLX Titanium Genome Sequencer (Roche/454 Life Sciences). Newbler de novo assembler software (Roche) was used for the sequence assembly. Final gap closure and sequence polishing were performed by capillary sequencing of PCR products using an ABI3730xl DNA Analyzer (Applied Biosystems).

### Bioinformatics

Bioinformatic characterization of the nucleotide sequence of pP62BP1 was performed using specialized software tools. The sequence was initially analyzed using ORF Finder (http://www.ncbi.nlm.nih.gov/gorf/gorf.html), Clone Manager (Sci-Ed8) and Artemis (Rutherford et al. 2000). Similarity searches were performed using the BLAST programs (Altschul et al. 1997), PRIAM (Claudel-Renard et al. 2003) and REBASE (Roberts et al. 2010). Helix-turn-helix motifs were identified by the application of GYM 2.0 (Gao et al. 1999; Narasimhan et al. 2002) and Helix-Turn-Helix Motif Prediction (Dodd and Egan 1990). Other conserved domains and motifs were identified using Motif Scan (Pagni et al. 2007), the NCBI Conserved Domain Database (CDD; Marchler-Bauer et al. 2011) and the Pfam database (Finn et al. 2010). Sequence alignments were performed using MUSCLE (Edgar 2004). Putative promoter sequences were predicted using BPROM (http://www.softberry.com/berry.html). Protein secondary structures were determined with the application of PredictProtein (Rost et al. 2003) and YASPIN (Lin et al. 2005). Protein signal sequences were identified using PrediSi (Hiller et al. 2004).

### Nucleotide sequence accession number

The nucleotide sequence of plasmid pP62BP1 determined in this study has been annotated and deposited in the GenBank database with the accession number JQ065022.

## Results and discussion

### Overall features of pP62BP1

**Fig. 1:**
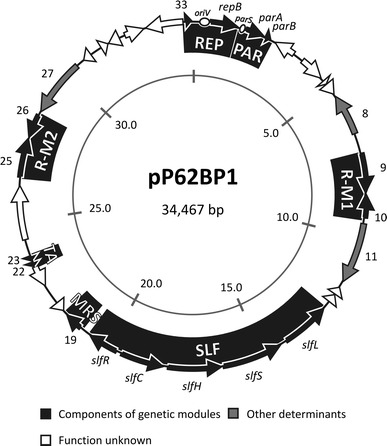
Circular representation of pP62BP1. Open reading frames (ORFs) are represented by *block arrows* on the *outer circle*. Predicted functions/homologies of the ORFs are indicated by the color key. Several ORF numbers and names are given for reference (see Table 1). The positions of the putative *oriV* and *parS* sequences are indicated by *white ellipses* (see text for details). *Solid black blocks* represent the genetic modules identified in the plasmid: REP, replication module; PAR, partitioning system; R-M1 and R-M2, restriction-modification systems; SLF, phenotypic module; MRS, multimer resolution system; TA, toxin–antitoxin system. The *inner circle* is a graduated scale (kb)

Plasmid pP62BP1 is the only extrachromosomal replicon residing in *Psychrobacter* sp. DAB_AL62B. The complete nucleotide sequence of pP62BP1 (34,467 bp) revealed the presence of 33 open reading frames (ORFs) (Fig. 1), which constitute 81.5% of the plasmid genome. In silico sequence analysis permitted the assignment of putative functions to 18 of the predicted ORFs. The remaining ORFs show the highest level of similarity (on the amino acid sequence level) to proteins of unknown function (12 ORFS), or have no relatives in the open databases (3 ORFs) (Table 1). The overall G+C content of the pP62BP1 nucleotide sequence (36.5%) is lower than the values determined for the plasmid or chromosomal DNAs of other *Psychrobacter* spp. strains (38.0–46.0%).

**Table 1:** Putative ORFs of pP62BP1 and their functions

ORF name	Position in sequence		Protein length (aa)	Predicted gene function	Most relevant homolog		
	Strand	bp			Number of identities/number examined (%)	Organism (plasmid)	GenBank accession number
*repB*	+	549–1,466	305	Initiator RepB protein	154/308 (50)	*Psychrobacter* sp*.* J466	ACY02903
*parA*	+	1,707–2,345	212	Partitioning protein ParA	203/211 (96)	*Psychrobacter cryohalolentis* K5 (plasmid 1)	ABE76262
*parB*	+	2,355–2,558	67	Partitioning protein ParB	39/68 (57)	*Enhydrobacter aerosaccus* SK60	EEV21786
ORF4	−	3,412–2,711	233	No significant similarity			
ORF5	+	3,755–4,567	270	Hypothetical protein	37/79 (47)	*Xanthomonas oryzae* pv*. oryzae* MAFF 311018	BAE70960
ORF6	+	4,681–4,815	44	Hypothetical protein	25/44 (57)	*Neisseria lactamica* Y92-1009	CBX21727
ORF7	+	4,824–5,219	131	Hypothetical protein	37/123 (30)	*Neisseria lactamica* Y92-1009	CBX21727
ORF8	−	6,634–5,303	443	RNA-directed DNA polymerase (reverse transcriptase)	267/483 (55)	*Psychrobacter* sp. 1501(2011)	EGK13519
ORF9	+	7,289–8,563	424	DNA (cytosine-5-)-methyltransferase	279/404 (69)	*Vibrio* sp. RC586	EEY99607
ORF10	−	9,345–8,560	261	Type II site-specific deoxyribonuclease	135/264 (51)	*Lachnospiraceae* bacterium 5_1_63FAA	EFV17523
ORF11	+	9,533–11,488	651	Putative DNA mismatch repair protein	406/656 (62)	*Acinetobacter baumannii* AB900	ZP_04661841
ORF12	−	11,957–11,697	86	Hypothetical protein	29/80 (36)	*Pastereulla dagmatis* ATCC 43325	EEX49811
ORF13	−	12,256–11,978	92	Hypothetical protein	32/90 (36)	*Pastereulla dagmatis* ATCC 43325	EEX49811
ORF14	−	14,108–12,483	541	Medium-chain-fatty-acid-CoA ligase	359/540 (66)	*Acinetobacter johnsonii* SH046	EEY97115
ORF15	−	16,274–14,307	655	Alkyl sulfatase	365/631 (58)	*Pseudomonas aeruginosa* 39016	EFQ36922
ORF16	−	18,034–16,274	586	GMC oxidoreductase family protein	434/565 (77)	*Acinetobacter johnsonii* SH046	EEY97634
ORF17	−	19,565–18,132	477	NAD-dependent aldehyde dehydrogenase	364/468 (78)	*Acinetobacter johnsonii* SH046	EEY97635
ORF18	+	19,732–20,751	339	AraC/XylS family transcriptional regulator	194/339 (57)	*Acinetobacter johnsonii* SH046	EEY97636
ORF19	+	21,088–21,720	210	Resolvase domain-containing protein	166/201 (83)	*Psychrobacter* sp*.* 1501(2011)	EGK07211
ORF20	−	22,172–21,852	106	Hypothetical protein	17/46 (37)	*Psychrobacter* sp*.* 1501(2011)	EGK13551
ORF21	−	23,486–22,821	229	Hypothetical protein	125/202 (62)	*Psychrobacter arcticus* 273-4	AAZ19228
ORF22	−	23,676–23,494	60	No significant similarity			
ORF23	−	23,887–23,699	62	Putative toxin of *hicAB* family	33/60 (55)	*Caldicellulosiruptor obsidiansis* OB47	ADL43536
ORF24	+	24,421–26,145	574	Hypothetical protein	123/564 (22)	*Pantoea* sp*.* AT-9b	ADU72791
ORF25	+	26,411–27,685	424	DNA (cytosine-5-)-methyltransferase	271/410 (66)	*Acinetobacter baumannii* ATCC 19606	EEX04797
ORF26	−	28,467–27,682	261	Type II site-specific deoxyribonuclease	135/264 (51)	*Lachnospiraceae* bacterium 5_1_63FAA	EFV17523
ORF27	−	30,452–28,593	619	SMC domain-containing protein	270/639 (42)	*Ilyobacter polytropus* 2926 (pILYOP01)	ADO84398
ORF28	−	31,016–30,726	96	No significant similarity			
ORF29	+	31,407–31,655	82	Hypothetical protein	20/45 (44)	*Acinetobacter baumanii* ATCC 19606	EEX02196
ORF30	−	32,055–31,681	124	Hypothetical protein	41/134 (31)	*Pantoea* sp*.* AT-9b	ADU68442
ORF31	+	32,253–32,894	213	Hypothetical protein	22/55 (40)	*Moraxella bovis* Epp63 (pMBO-2)	BAD83748
ORF32	−	34,151–32,946	401	Hypothetical protein	86/386 (22)	*Hahella chejuensis* KCTC 2396	ABC32288
ORF33	+	34,210–34,443	78	XRE family transcriptional regulator	42/66 (64)	*Vibrio cholerae* 12129(1)	EEO01051

Comparative sequence analysis identified several putative genetic modules within the pP62BP1 genome. The plasmid backbone is composed of a replication system (REP) and three putative stabilization systems, responsible for active partitioning (PAR), resolution of multimeric plasmid forms (MRS) and postsegregational elimination of plasmid-less cells from the bacterial population (toxin–antitoxin system, TA). The additional genetic load includes (a) two highly related restriction-modification systems (R-M) (ORF9-ORF10 and ORF25-ORF26), (b) a putative novel catabolic operon and its regulator (SLF module), and (c) a putative retroelement (ORF8).

### Replication system of pP62BP1

The replication systems (REP) of the vast majority of plasmids residing in gram-negative bacteria consist of (a) a gene encoding a replication initiation protein (Rep) and (b) a *cis*-required origin site (*oriV*), often containing iterons, i.e. directly repeated sequences representing Rep protein binding sites. Both of these elements could be distinguished within the REP region of pP62BP1, which has a structure typical for many theta-replicating plasmids.

The predicted RepB protein of pP62BP1 (ORF1; 305 amino acids; aa) shows the highest level of aa sequence identity (50%) with replication initiation proteins encoded by two *Psychrobacter* sp. strains, J466 and 1501(2011), isolated from the gut of the Atlantic herring (*Clupea harengus*) (Curson et al. 2010) and human blood, respectively (GenBank acc. nos. ACY02903 and EGK07200). A significant level of sequence similarity is also shared with a hypothetical protein (46%) of *Kingella oralis* ATCC 51147 (*Betaproteobacteria*; family *Neisseriaceae*) (GenBank acc. no. EEP66524) and replication proteins (30–35%) of several plasmids of *Gammaproteobacteria*: (a) p2ABSDF from *Acinetobacter baumanii* SDF (CAP02944), (b) pMMCU1 of *Acinetobacter calcoaceticus* (ACT83384), (c) pVSAL43 of *Aliivibrio salmonicida* (LFI1238; CAQ76621), (d) pALVIN01 of *Allochromatium vinosum* DSM 180 (ADC64139) and (e) pMBO-1 of *Moraxella bovis* Epp63 (BAD83730).

Inspection of the primary structure of the putative pP62BP1 RepB protein and its two closest homologs revealed the presence of two analogously located conserved regions, containing (a) a helix-turn-helix DNA binding motif (HTH) (residues 170–190 in RepB) (Dodd and Egan 1990) and (b) an N-terminal leucine zipper-like (LZ-like) motif that is probably responsible for dimerization of the Rep molecules (del Solar et al. 1998). The LZ-like motif of pP62BP1 RepB protein comprises 4 leucine residues separated by heptad intervals (Leu4, 11, 18, and 25).

Both the HTH and LZ-like motifs are also conserved in the well-characterized RepA protein of plasmid pPS10 from *Pseudomonas syringae* pv. *savastonoi* (Giraldo and Fernández-Tresguerres 2004), which shares around 32% identity with the putative polypeptide product of pP62BP1 ORF1. Analysis of the crystal structure of pPS10 RepA revealed the presence of two “winged-helix” domains (WH1 and WH2) (Giraldo et al. 2003), which play a crucial role in the control of initiation of plasmid replication (Gasset-Rosa et al. 2008). In silico analysis of the predicted secondary structure of the RepB of pP62BP1 (PredictProtein, YASPIN) suggests that this protein contains analogous WH domains in the regions covered by residues 45–120 and 125–230 (data not shown).

**Fig. 2:**
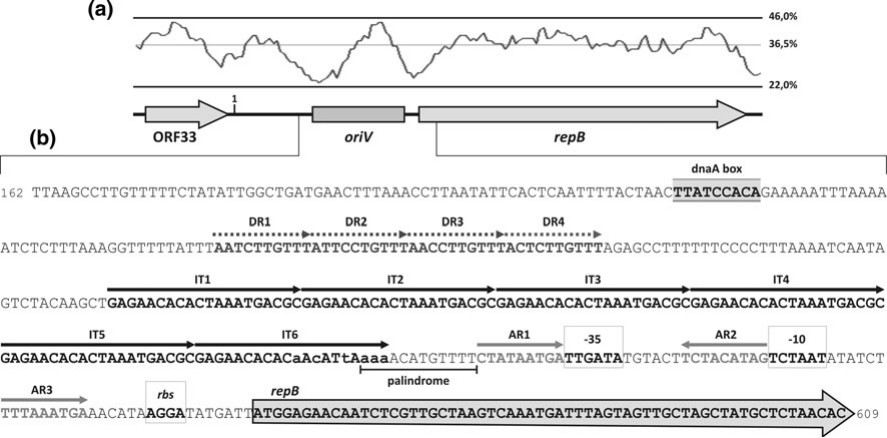
**a** Structure of the REP module of pP62BP1. A G+C content profile is shown in the *upper panel* with the relative positions of ORF33, *oriV* and *repB* indicated below (*1* the arbitrary starting point of the plasmid’s nucleotide sequence). **b** Sequence organization of the proposed *oriV*. The dnaA box is *shaded*. *Dashed arrows* indicate A+T-rich sequences (DR1–DR4). Six iteron sequences (IT1–IT6) are marked by *black arrows*. The non-identical residues in the IT6 sequence are written in *lowercase*. Palindromic sequence is *underlined*. Additional repeats similar to the 3′-part of iterons (AR1–AR3) are indicated by *gray arrows*. The *repB* −35 and −10 promoter elements and a potential ribosome binding site (*rbs*) are *boxed*. The coding sequence of *repB* is marked by a *large gray* *arrow* in the background

The putative *oriV* of pP62BP1 is located immediately upstream of *repB* (Fig. 2). This region contains six tandem repeat sequences of 20 bp (putative iterons, designated IT1–IT6), which begin 197 bases upstream of the predicted start codon (ATG) of *repB*. Iterons IT1–IT5 are identical, while IT6 differs from the others by 6 bases. It is noteworthy that the three 3′-terminal bases of IT6 are located within one of the inverted repeats that form a 12-bp-long palindromic sequence (Fig. 2b).

The iteron-containing region of pP62BP1 (120 bp) is relatively G+C-rich (~42%) and is surrounded by two A+T-rich DNA segments (Fig. 2a). The one situated upstream of the iterons contains four adjacent A+T-rich direct repeats (DR1-DR4; consensus: 5′-ANT/CCT/CTGTTT-3′), which may function as a strand melting site during the initiation of plasmid replication (Fig. 2b). Upstream of DR1 there is a short sequence element (5′-TTATCCACA-3′) matching the consensus sequence recognized by a chromosomally encoded protein DnaA (5′-TTA/TTNCACA-3′; Messer 2002). The DnaA protein has been shown to participate in the replication initiation of several iteron-containing plasmids (del Solar et al. 1998). The A+T-rich region downstream of the iterons carries a predicted promoter sequence for *repB*. Its −35 box is surrounded by two imperfect inverted repeats, designated as AR (additional repeats; AR1: 5′-CTATAATGA-3′ and AR2: 5′-CTATGTAGA-3′), which share some similarity with the 3′-termini of the iterons (5′-CTAAATGACGC-3′). A third sequence of this type (AR3: 5′-TTTAAATGA-3′) was identified between the −10 TATA box and a putative ribosome binding site (*rbs*) of *repB* (Fig. 2b).

It is noteworthy that the organization of the putative *oriV* of pP62BP1 resembles that of pPS10, although the level of nucleotide sequence identity of these regions is rather low (data not shown). Plasmid pPS10 is a model iteron-containing, theta-replicating plasmid, whose replication has been thoroughly studied (Giraldo and Fernández-Tresguerres 2004). Based on the observed similarity between the Rep proteins and the shared structure of the *oriV* regions of pP62BP1 and pPS10, we assume that the replication control of these plasmids functions in a similar way, i.e. by binding of Rep proteins to the iterons or AR sequences, depending on the conformation of the proteins.

In contrast to pPS10, plasmid pP62BP1 contains an additional ORF (ORF33) which may constitute part of the REP region (Fig. 2a). ORF33 is located upstream of the putative *oriV* of pP62BP1 and predicted to encode a 69-aa protein containing a conserved HTH_XRE DNA binding domain (cd00093; residues 23–44) commonly found in many bacterial transcriptional regulators, including a number of TA system antitoxin proteins. The amino acid sequence of the predicted ORF33 protein shares the highest level of similarity with polypeptides encoded by several gammaproteobacteria, e.g. *Vibrio cholerae* 12129(1) and *Enhydrobacter aerosaccus* SK60 (64% identity; GenBank acc. nos. EEO01051 and EEV22138, respectively).

It is noteworthy that a protein of the XRE family (named RepA), which is highly related to ORF33, is also encoded within the REP region of the broad host range plasmid RA3 (IncU group). As in pP62BP1, the RA3 *repA* gene is located upstream of the ORF encoding the replication initiation protein RepB (which is not related to the Rep protein of pP62BP1). In the case of RA3, it has been demonstrated that expression of the *repA* gene is repressed by both the RepA and RepB proteins, which strongly suggests that the *repA* promoter can influence the regulation of *repB* expression (Kulinska et al. 2008). We hypothesize that a similar regulatory circuit may also occur in pP62BP1, but this has to be experimentally confirmed.

### Stable maintenance systems

#### Partitioning system

Immediately upstream of the pP62BP1 REP module, two ORFs (ORF2 and ORF3) representing a putative partitioning system (PAR) were identified. Plasmid-encoded PAR systems, responsible for proper segregation of plasmid copies to daughter cells at cell division, are usually composed of two genes (often termed *parA* and *parB*) and a partitioning site where the ParB protein binds (Ebersbach and Gerdes 2005).

The predicted product of ORF2 contains a conserved ParA domain (cd02042) with a deviant Walker-A type ATPase motif adjacent to its N terminus (KGGSKGT, residues 10–16). The closest homologs of the ORF2 protein are encoded by genes in plasmid 1 of *P. cryohalolentis* K5 (97% identity) and in the chromosome of *Enhydrobacter aerosaccus* SK60 (80% identity).

ORF3 is placed downstream of ORF2 and encodes a putative protein with a ParG domain (PF09274). Moreover, the predicted secondary structure of the potential ORF3 product corresponds to the *ribbon*-*helix*-*helix* structure confirmed for the ParG domain-containing protein of plasmid pTP228 (Golovanov et al. 2003). Database searches revealed that the two most similar proteins (87 and 57% identity, respectively) are encoded by ORFs situated immediately downstream of the aforementioned ORF2 homologs from plasmid 1 of *P. cryohalolentis* K5 and *E. aerosaccus*. However, it is noteworthy that the coding sequence corresponding to ORF3 was not distinguished during annotation of the *P. cryohalolentis* K5 plasmid 1 sequence deposited in the GenBank database (acc. no. CP000324, position 21,128–21,331).

Taking into consideration (a) the predicted length of the polypeptides encoded within the *par* locus of pP62BP1, (b) the presence of a Walker-A type motif in the putative ParA (and absence of a HTH motif), and (c) the weak conservation of a predicted ParB aa sequence, it may be concluded that ORF2 and ORF3 represent the *parA* and *parB* genes of a type Ib partitioning system (Ebersbach and Gerdes 2005). The genes are separated by a short intergenic sequence (9 bp) and both are preceded by putative ribosome binding sites. Since the *rbs* of *parB* partially overlaps the stop codon of *parA*, it is highly probable that the two genes constitute an operon.

The third component of the putative type Ib *par* locus of pP62BP1, i.e. a centromere-like sequence *parS*, is thought to be located between the −10 TATA box and *rbs* of *parA*. This region is relatively A+T-rich and contains seventeen identical, adjacent 8-bp direct repeats (5′-AATACTCA-3′). Comparative analyses of nucleotide sequences upstream of genes encoding proteins with homology to ParA revealed that such sequence organization is apparently unique for pP62BP1. Interestingly, we found twelve 5′-AATACTCA-3′ repeats in the sequence of plasmid 1 of *P. cryohalolentis* K5. However, these repeats are not adjacent to the *parA* homolog, but form the 3′ terminus of a putative protein coding sequence Pcryo_2479 (see below).

It is also noteworthy that a stretch of eight 5′-TTTACTCA-3′ repeats similar to those present in the putative *parS* site of pP62BP1 was identified within the intergenic region between ORF27 and ORF28 (residues 31,259–31,298 bp). However, it is not known whether ParB binds to these sequences.

#### Toxin–antitoxin system

ORF23, one of the smallest among the identified coding sequences of pP62BP1, encodes a putative 62-aa polypeptide that belongs to the family of predicted HicA-like RNA binding proteins (COG1724). These proteins are thought to function as toxins of *hicAB* addiction systems, which are responsible for post-segregational elimination of plasmid-less cells from a bacterial population (Makarova et al. 2006; Jørgensen et al. 2009). The closest homologs of the ORF23 product (48–55% of identity) were identified in *Caldicellulosiruptor obsidiansis* OB47 (GenBank acc. no. ADL43535), *C. saccharolyticus* DSM 8903 (ABP65736) and *Burkholderia xenovorans* LB400 (ABE32795). Amino acid sequence alignments of these proteins gave a consensus which matches that for HicA-like proteins reported by Makarova et al. (2006). It is noteworthy that downstream of ORF23 we identified a short ORF22 encoding a putative 60-aa protein without any significant similarity to sequences deposited in open databases. However, corresponding short ORFs were also identified downstream of the aforementioned ORF23 homologs and their sequence alignment confirms some level of similarity to the HicB antitoxin consensus sequence (data not shown). Further analysis is required to determine whether ORF22 and ORF23 of pP62BP1 constitute a functional TA system.

#### Multimer resolution system

The amino acid sequence of the deduced ORF19 protein was found to contain a catalytic domain characteristic of the superfamily of serine recombinases (SR) which perform site-specific recombination of DNA molecules (cd00338). The enzymes of this group are functionally versatile and include integrases, resolvases, transposases and invertases (Smith and Thorpe 2002). The pP62BP1-encoded SR shares the highest level of similarity with a subgroup of these proteins harboring a SR_rep_par domain (cd03767). Typically, they function as resolvases in multimer resolution systems which represent a type of plasmid stabilization mechanism (Bahl et al. 2009).

Four amino acid sequences that exhibit more than 80% identity to the described SR were identified in *Psychrobacter* sp. 1501(2011) (GenBank acc. no. EGK07211), plasmid pRWF101 of *Psychrobacter* sp. PRwf-1 (ABQ95337), and in two strains of *Acinetobacter baumanii:* 6013150 (EGJ69339) and 6013113 (EGJ65601). Interestingly, careful analysis of the nucleotide sequence of plasmid 1 of *P. cryohalolentis* K5 identified two adjacent coding sequences whose products, designated Pcryo_2509 (ABE76286) and Pcryo_2479 (ABE76256), are similar to the N- and C-terminal parts of the pP62BP1-encoded SR, respectively. In silico joint translation of these two ORFs (including the intergenic sequence) produced an amino acid sequence 84% identical to that of the pP62BP1 ORF19 product (data not shown). We assume that a single transversion 5′-GAA-3′ → 5′-TAA-3′ resulting in a Glu → STOP_*ochre*_ nonsense mutation might have been responsible for disrupting the original ORF19 homolog in the strain K5 plasmid. Moreover, in the 3′-terminal region of the Pcryo_2479 coding sequence, we discovered, to our surprise, 12 repeated sequences identical to those forming the putative *parS* of the pP62BP1 partitioning system (see above).

It is noteworthy that ORF19 is flanked by 83-bp-long inverted repeats, IRL_RES_ and IRR_RES_, that are 77% identical (different in 20 nt positions). IRL_RES_ ends 22 nt upstream of the gene’s putative START codon, while IRR_RES_ contains its termination codon. Comparative analyses confirmed that similar sequences occur in several replicons of *Psychrobacter* spp., including the chromosomes of *P. cryohalolentis* K5, *P. arcticus* 273-4 and two *Psychrobacter* sp. strains, 1501(2011) and PRwf-1; while in the aforementioned plasmid 1 of *P. cryohalolentis* and pRWF101, single copies of the IR are found upstream of the ORFs encoding the pP62BP1 SR homologs.

### Additional genetic load

#### Restriction-modification systems

Sequences encoding two restriction-modification systems, designated R-M1 (ORF9-ORF10) and R-M2 (ORF25-ORF26), were identified in pP62BP1. Both consist of genes encoding a methyltransferase (MTase) and a restriction endonuclease (REase) placed tail to tail with overlapping termination codons. Interestingly, these two systems (separated by ca. 17 kbp) show a very high level of identity, since the nucleotide sequences of their corresponding components differ only in 33 nt (MTase genes) and 16 nt (REase genes). Consequently, the level of amino acid identity of the potential MTases is 99% (420/424 aa), and that of the putative REases is 96% (250/261 aa).

Sequence comparisons revealed that the putative MTases encoded by ORF9 and ORF25 are highly similar to numerous C5-cytosine-specific DNA methylases, encoded by, e.g. *Vibrio* sp. RC586 (69% identity; GenBank acc. no. EEY99607), *Acinetobacter baumanii* ATCC 19606 (66%; EEX04797), and *Lactococcus lactis* subsp. *cremoris* UC503 (55%; AAB66696). The recognition sequence of these enzymes is predicted to be 5′-CCNGG-3′ (methylated cytosine underlined).

The predicted amino acid sequences of the protein products of ORF9 and ORF25 contain six motifs conserved among C5-methyltransferases (Pósfai et al. 1989) (data not shown). Sequence conservation of the potential target recognition domains (TRDs) of the pP62BP1-encoded MTases and their homologs indicates that they are likely to recognize the same 5′-CCNGG-3′ sequence. Furthermore, a MUSCLE alignment of 93 characterized and putative cytosine C5-methyltransferases with the same recognition sequence deposited in REBASE (data not shown) suggested that conservation of the amino acid string G(K/N)GFx_13_T(I/L)x_3_YYK in their TRDs may be characteristic of 5′-CCNGG-3′ MTases (cf. Neely and Roberts 2008). This motif corresponds to residues 316–340 in the ORF9 and ORF25 products. Moreover, within the N-terminal regions of these proteins, we identified a HTH_XRE domain (cd00093), which may be responsible for DNA–protein interactions other than the recognition of a methylation site, as was experimentally proved for the aforementioned enzyme M.ScrFI of *Lactococcus lactis* subsp. *cremoris* UC503 (Butler and Fitzgerald 2001).

The potential proteins encoded by ORF10 and ORF26 belong to the LlamI family of type II REases and so are predicted to cleave DNA after the first cytosine in the sequence 5′-CCNGG-3′. Only five proteins with significant homology to the pP62BP1 REases were found in the open databases. All of these are encoded in the vicinity of the aforementioned MTase homolog genes in *Lachnospiraceae* bacterium 5_1_63FAA (51% identity; GenBank acc. no. EFV17523), *V. parahaemolyticus* AQ4037 (48%; EFO45071), *P. marinus* NATL1A (46%; ABM74602), and *L. lactis* subsp*. cremoris* strains U503 (46%; AAB66695) and M19 (45%; AAM03111) [NB. the methyltransferase sequences of these two strains are identical (Szatmari et al. 2006)]. In the amino acid sequences of all these putative REases we identified a bipartite catalytic motif PD–(D/E)XK (residues 56–64 in the pP62BP1 REases) (Orlowski and Bujnicki 2008). Notably, the 4-aa-long spacer sequence between the conserved PD and (D/E)XK amino acid strings is unusually short (Kosinski et al. 2007).

The amino acid sequences of two of the aforementioned REases from *L. lactis* were used by Kosinski et al. (2007) to construct a protein–DNA interaction model of the R.*Mva*I enzyme, which was partially confirmed following determination of the crystal structure of this complex (PDB: 2OA9_A) (Kaus-Drobek et al. 2007). We performed a comparative analysis of the amino acid sequences of R.*Mva*I and pP62BP1 REases and, in spite of the low level of sequence identity (13% identity, 34/241 aa), analogies in the secondary structures of these proteins were observed. Moreover, seven amino acids of proven importance to the functionality of R.*Mva*I are conserved in the ORF10 and ORF26 products. These residues presumably take part in (a) the interaction with the phosphate backbone of DNA (residues K101, K183), (b) the recognition of C and G residues within the restriction site (D226, R241), and (c) the complexing of Mg^2+^ in the catalytic site of the enzyme (D57, E62, K64).

**Fig. 3:**
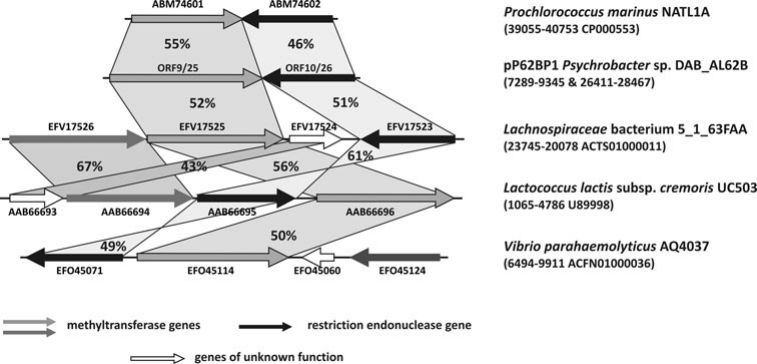
Comparison of the genetic structures of R-M systems encoding proteins homologous to the enzymes of the R-M systems harbored by pP62BP1. Organism names, the ranges of the compared sequences and their GenBank acc. nos. are shown on the *right*. (NB Only one genetic structure is shown for pP62BP1, representing both R-M1 and R-M2.) ORFs are marked by *block arrows* colored according to the proposed function of their product (see legend). The GenBank acc. nos. of the encoded proteins are given above or below each ORF. The *percentage values* indicate the level of identity between proteins encoded by corresponding ORFs, which are paired by *shaded regions*

The aforementioned tail to tail organization of the ORFs of the pP62BP1 R-M1 and R-M2 systems with overlapping of their 3′ ends is rather uncommon among the restriction-modification systems (Wilson and Murray 1991). A comparison with the four most closely related systems described above revealed that tail to tail orientation of the component genes exists only in the case of *P. marinus* NATL1A (Fig. 3). Each of the three other homologous systems contains an additional methyltransferase gene as well as an ORF encoding a putative protein of unknown function (COG4933), neither of which is present in pP62BP1 or *P. marinus*.

#### *The putative* slf *operon*

**Fig. 4:**
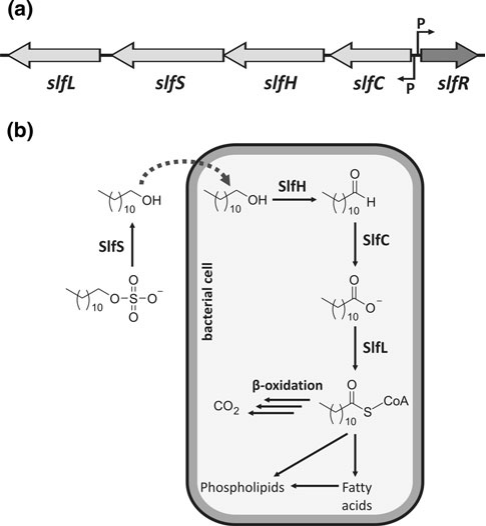
**a** Genetic organization of the pP62BP1 SLF module. ORFs are represented by *block arrows*. The location and orientation of predicted promoter sequences (*P*) for *slfC* and *slfR* are indicated by *arrows*. **b** Proposed metabolic pathway comprising reactions catalyzed by the enzymes encoded in the *slf* operon, with dodecyl sulfate as the starting substrate (see text for details). *Dashed arrow* indicates transport into the cell. The proposed enzyme names are based on their functions: SlfS, alkyl sulfatase; SlfH and SlfC, dehydrogenases catalyzing oxidation of hydroxyl and carbonyl groups, respectively; SlfL, fatty-acid-CoA ligase

The region of the pP62BP1 sequence located between R-M1 and R-M2 was found to contain a putative phenotypic module comprising nearly 25% of the plasmid genome. This is composed of five ORFs, four of which (*slfL, slfS, slfH, slfC*) are tandemly oriented, with the fifth (*slfR*) located upstream of the others in the reverse transcriptional orientation (Fig. 4a). Analysis of the possible functions of the encoded proteins suggested that they may enable the bacterial host to metabolize organic sulfates present in the environment.

The crucial enzyme in this process—an alkylsulfatase—is a putative product of the ORF designated *slfS*. Sulfatases (EC 3.1.6-) constitute a large and heterogeneous group of enzymes which catalyze the hydrolysis of organic esters of sulfuric acid to produce the corresponding alcohol and hydrogen sulfate (Hanson et al. 2004). Three types of sulfatases have been distinguished to date: (1) predominantly eukaryotic arylsulfatases, (2) sulfatases belonging to the Fe^2+^ α-ketoglutarate-dependent dioxygenase superfamily, and (3) sulfatases related to metallo-β-lactamases (Long et al. 2011). Comparative analysis allowed us to classify SlfS as a type 3 sulfatase. The putative protein shares extensive amino acid sequence homology with numerous enzymes of *Pseudomonas* spp; in particular, a considerable level of identity (57%) was observed with the well-characterized SdsA1 of *P.* *aeruginosa* PAO1 (Hagelueken et al. 2006). Amino acid sequence alignment of SlfS and SdsA1 revealed their similar domain organization (data not shown). A 21-aa-long signal peptide was identified at the N terminus of SlfS (PrediSi), which corresponds to the 19-aa sequence of SdsA1 that was shown to direct secretion of this enzyme. Both proteins harbor (a) a Zn^2+^-binding motif (T**H**x**H**x**DH**x**GG**x_102_ **E**x_18_A**E**x_44_ **H**), which forms part of a metallo-β-lactamase-like domain, (b) a C-terminal SCP-2 domain, and (c) a central dimerization domain.

The four other ORFs of the pP62BP1 SLF module encode putative proteins whose closest homologs were found in *Acinetobacter johnsonii* SH046 (see Table 1). Detailed bioinformatic analyses identified the *slfL* product as a fatty-acid-CoA synthetase (FACS; EC 6.2.1.3), which belongs to the adenylate-forming enzyme superfamily (Black and DiRusso 2003). Two characteristic motifs were found in this protein: an ATP-AMP motif YTSGTTGxPKGVx_125-130_GYGxTE, conserved in all the enzymes of the superfamily and a FACS signature motif DGWLHTGDIGxWxPxGxLKIIDRKK (Black et al. 1997). In silico analyses indicated that SlfL is closely related to the FACS enzymes specific for long- or medium-chain fatty acids (NCBI Conserved Domains Search). The ligation of these substrates with CoA, presumably catalyzed by SlfL, results in the production of acyl-CoA, which may subsequently become a substrate for β-oxidation or the biosynthesis of other lipids.

The segment of the SLF module that contains *slfH*, *slfC* and *slfR* was found to be equivalent to a region of the *A. johnsonii* SH046 chromosome (see Table 1). The conserved domains GMC_oxred_N and GMC_oxred_C, which are characteristic of GMC oxidoreductases, were identified in the deduced amino acid sequence of SlfH (Cavener 1992). Since the GMC_oxred_N domain binds FAD as a cofactor, these enzymes appear to belong to the flavoprotein superfamily (Fraaije and Mattevi 2000). The two domains are also found in long-chain alcohol dehydrogenases (EC 1.1.3.20), so SlfH might catalyze their oxidation to the corresponding aldehyde. The putative SlfC protein is predicted to exhibit similar oxidoreductase activity, although with specificity for aldehydes (Pfam: PF00171). This enzyme is likely to require NAD^+^ or NADP^+^ as a cofactor.

We propose a chain of reactions catalyzed by the four identified enzymes leading from alkylsulfate to acyl-CoA (Fig. 4). Secreted SlfS hydrolyses the alkyl sulfate and the produced alcohol (after being transported into the bacterial cell) is oxidized to a fatty acid, which, in turn, is activated by SlfL-mediated ligation with CoA. The presence of a similar metabolic pathway for the biodegradation of dodecyl sulfate (SDS) by *Pseudomonas* sp. C12B was confirmed by Thomas and White (1989). Using the ^14^C-radiotracer technique they observed that ~70% of the radiolabel was released as ^14^CO_2_ as a result of β-oxidation, while the remainder was incorporated into cellular components such as membrane lipids.

The exact nature of the starting substrate for the proposed pathway remains an open question. In a preliminary experiment we confirmed that *Psychrobacter* sp. DAB_AL62B can use SDS as the sole source of carbon and energy (data not shown). However, it seems unlikely that this anthropogenic compound represents the main substrate for alkylsulfatase in the Arctic environment, unless it is a contaminant from scientific research, the fishing industry or tourism (George 2002). Hagelueken et al. (2006) suggested that the specificity of SdsA1 (a homolog of the pP62BP1-encoded SlfS) towards SDS is incidental and that sulfated sugars such as sulfoglycolipids may represent its physiological substrates. Such compounds have been detected in the salt glands of sea birds (Ishizuka 1997), so it is not unlikely that they are present in the environment from which the pP62BP1 host strain was isolated. Furthermore, the ability to directly use alkyl chains in the synthesis of membrane elements, confirmed by Thomas and White (1989), may be of adaptive importance to psychrophilic bacteria. It is known that the modulation of fatty acid composition is a common mechanism for maintaining the correct membrane fluidity at low temperatures (Wang et al. 2009).

The genetic organization of the region containing the ORFs encoding the aforementioned enzymes suggests that they are arranged in an operon. It is noteworthy that although the reactions comprising the proposed metabolic pathway have been long known, we are unaware of any previous reports of a similarly structured gene cluster. In addition, we predict that the activity of the operon is controlled by a transcriptional regulator SlfR. This protein belongs to the AraC/XylS family that includes a large number of activators involved in carbon metabolism, stress response and virulence (Egan 2002). The conserved domains Arabinose_bd (PF00165) and HTH_AraC (PF12625) were identified in the N- and C-terminal regions of SlfR, respectively. The latter contains two helix-turn-helix motifs characteristic of AraC/XylS family proteins, while the N-terminal domain is most probably responsible for effector binding and dimerization of the regulator (Domínguez-Cuevas et al. 2008).

Typically, the mechanism of transcription control by AraC/XylS family regulators involves binding to specific DNA sequences within cognate promoters, which results in positive stimulation of gene expression. This depends on the recognition of a specific effector that causes a conformational change in the regulator and consequently its correct positioning at the binding sites (Gallegos et al. 1997). We assume that the identified SlfR protein may interact with series of sequence repeats which overlap the putative promoters of *slfC* and *slfR*. Interestingly, among these we identified a pair of direct repeats, 5′-TTGA(N_6_)AGGACA-3′, which have a similar structure to the XylS binding sites (Domínguez-Cuevas et al. 2010). Corresponding repeated sequences are also present upstream of ORFs encoding the closest homologs of SlfR from *A. johnsonii* SH046 (GenBank acc. no. EEY97636), *Aliivibrio salmonicida* LFI1238 (CAQ81021) and *Shewanella* sp. ANA-3 (ABK46545).

The organization of the intergenic sequence between *slfC* and *slfR* suggests that the proposed binding of the regulator may affect transcription from both promoters. According to our prediction, the mechanisms of SlfR activity may resemble the model proposed for *Pseudomonas putida* XylS protein (Domínguez-Cuevas et al. 2008). If this is the case, SlfR should bind to specific sequences in the presence of an effector and thus promote the transcription from the *slfC* promoter, possibly simultaneously autoregulating its own expression. Functional analysis will be necessary to elucidate the exact nature of this process, including identification of the effector molecule and examination of the possible existence of secondary promoters or other regulatory sequences.

#### Other ORFs

More than 50% of the ORFs of pP62BP1 do not belong to the aforementioned genetic modules (see Table 1; Fig. 1). Observed sequence similarities allowed us to predict the functions of putative proteins encoded by only three of the ORFs (ORF8, ORF11, and ORF26).

Detailed analysis of the amino acid sequence of the deduced ORF8 polypeptide suggested that it is related to reverse transcriptases (RT) of the RVT_1 family (PF00078), most often encoded by retroelements such as retrons or group II introns (Lampson et al. 2005). Within the putative protein sequence we identified seven conserved domains characteristic of RT (Xiong and Eickbush 1990), including domain 5 that contains a YADD motif comprising the enzyme active site (residues 281–284; Zimmerly et al. 2001). The presence of this ORF suggests that pP62BP1 carries a retroelement, most probably a retron.

In the N-terminal part of the putative 651-aa-long product of ORF11 we found a relatively short region (residues 23–69) with homology to the consensus sequence of the conserved domain of MutL-like proteins (COG0323). Members of this large protein family are involved in DNA mismatch repair and have been found in all domains of life (Yang 2000). However, the aforementioned limited homology is insufficient to assume a similar function for the ORF11 product.

In the sequence of the putative ORF27 polypeptide we found several motifs which suggest ATPase activity, including a Walker A motif in its N-terminal region, a Walker B motif, a D-loop and an H-loop in its central region. These features occur in a large group of proteins which harbor ATP-binding cassettes (ABC; cd00267). This superfamily comprises, among others, ABC transporters, DNA repair proteins (e.g. UvrA, MutS and Rad50) and proteins involved in the structural maintenance of chromosomes (SMC) (Hopfner and Tainer 2003). Bioinformatic analyses of the amino acid sequence encoded by ORF27 distinguished extensive overlapping segments that exhibit significant similarity to the conserved domains of OLD-type endonucleases and SMC proteins. The former enzymes are engaged in DNA replication, recombination and repair (COG3593), while the latter proteins are crucial for chromosome dynamics including their condensation and segregation during cell division (Graumann and Knust 2009). In the absence of supporting experimental evidence, it is not possible to predict the function of the putative ORF27 protein.

### Comparative analysis of pP62BP1 and other plasmids of *Psychrobacter* spp.

**Fig. 5:**
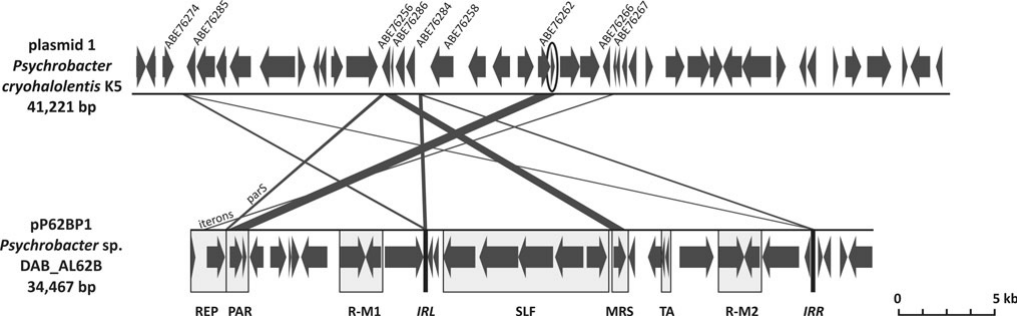
Linear alignment of pP62BP1 and plasmid 1 of *Psychrobacter cryohalolentis* K5. ORFs are marked by *block arrows.* In the strain K5 plasmid, several GenBank acc. nos. are given for reference, while the ORF whose sequence has not been deposited in GenBank is *encircled*. In pP62BP1, the defined genetic modules are *boxed* (see Fig. 1 for the abbreviations used). *Vertical black bars* mark the positions of 142-nt-long inverted repeats (IRL and IRR; see text for details). Regions of the plasmids sharing >70% identity (*E* value >1e−10) are connected by *black connecting lines*; those corresponding to pP62BP1 iterons and the *parS* sequence are marked accordingly. The scale is indicated by the *bar* in the *lower right-hand corner*

A comparative analysis of the nucleotide sequences of pP62BP1 and other plasmids of *Psychrobacter* spp. revealed significant levels of identity between certain sequence segments only in the case of the two largest plasmids known to date: plasmid 1 of *P. cryohalolentis* K5 (Fig. 5) and pRWF-101 of *Psychrobacter* sp. PRwf-1.

Highly similar ORFs presumed to encode serine recombinases (putative MRS systems) are present in all three replicons. However, as stated above, the sequence from the strain K5 plasmid appears to be disrupted and considerably rearranged, since the region corresponding to pP62BP1 *parS* is situated at its 3′ terminus. The presence of homologous PAR loci and iteron repeats in both of these replicons indicates some relationship between them, although it appears to have been obscured by genetic rearrangements that occurred in the evolution of the *P. cryohalolentis* K5 plasmid.

Other corresponding sequences are found within two regions of pP62BP1 which encompass a pair of 142-bp-long inverted repeats (80% identity; positions 11,496–11,637 and 31,055–31,196) (Fig. 5). Since similar sequences are also present in the chromosomes of *Psychrobacter* sp. PRwf-1, *P. cryohalolentis* K5 and *P. arcticus* 273-4, we assume that they might be associated with site-specific recombination or lateral gene transfer events that could have influenced the evolution of *Psychrobacter* spp. replicons.

## Conclusions

To date, pP62BP1 is the largest plasmid of *Psychrobacter* spp. for which a comprehensive analysis of its nucleotide sequence has been reported. In this study, we have used in silico methods to describe in detail the structure and putative functions of the genetic modules carried by this plasmid. The possible regulatory mechanisms of genes comprising several of these modules have also been proposed. We found that the compact backbone region of pP62BP1 consists of genes and regulatory sequences associated with its replication and stable inheritance (REP and PAR). Preliminary studies have confirmed that these modules are functional in other *Psychrobacter* spp. strains (data not shown), and we predict that they could be used to develop shuttle vectors for bacteria of this genus.

The two nearly identical restriction-modification systems harbored by pP62BP1 seem to be a feature that is unique among bacterial plasmids analyzed so far. There are two plausible non-mutually exclusive reasons why both R-M1 and R-M2 are stably maintained in this plasmid. First, they may function as a type of postsegregational cell killing system that increases stability of the plasmid in the bacterial population (Ichige and Kobayashi 2005). Second, a recombination event leading to the loss of one system or the production of a hybrid system could destabilize the putative common regulatory circuit controlling the expression of the R-M1- and R-M2-encoded genes. Further functional analyses of this plasmid may help to increase our understanding of this R-M regulatory mechanism and the role of these systems in prokaryotic genomes.

Likewise, a putative catabolic operon responsible for the transformation of organic sulfate esters, similar to that carried by pP62BP1, has not been described before. It is likely that possession of this operon allows the host strain to adapt to environmental conditions, presumably by conferring the ability to utilize a specific class of compounds for energy production and/or biosyntheses. The regulatory elements that stimulate the transcriptional activity of the *slf* operon have the potential to be used in the construction of vectors for a novel inducible protein expression system.

In summary, the findings of this study greatly expand our knowledge of plasmids of the psychrophilic bacteria as well as their possible role in the lateral gene transfer and the adaptation of microorganisms to specific Arctic environments. The presented analyses identify a number of interesting avenues to be explored in further experimental studies.
